# Predictive Value of Quantitative Parameters of ^18^F-FDG PET/CT in Patients with Liposarcoma

**DOI:** 10.3390/diagnostics14182021

**Published:** 2024-09-12

**Authors:** Lucia Martiniova, Serageldin Kamel, Kalevi Kairemo, Robert Benjamin, Neeta Somaiah, Gregory Ravizzini, Elise F. Nassif Haddad

**Affiliations:** 1 Department of Experimental Therapeutics, The University of Texas MD Anderson Cancer Center, Houston, TX 77054, USA; lmartini@mdanderson.org; 2 Radiation Oncology Department, The University of Texas MD Anderson Cancer Center, Houston, TX 77054, USA; serageddein@gmail.com; 3 Department of Theragnostics, Docrates Cancer Center, 00180 Helsinki, Finland; kalevi.kairemo@gmail.com; 4 Department of Nuclear Medicine, The University of Texas MD Anderson Cancer Center, Houston, TX 77030, USA; gravizzini@mdanderson.org; 5 Department of Sarcoma Medical Oncology, The University of Texas MD Anderson Cancer Center, Houston, TX 77030, USA; rbenjami@mdanderson.org (R.B.); nsomaiah@mdanderson.org (N.S.)

**Keywords:** F-18 FDG, PET/CT, outcomes, prediction, liposarcoma, segmentation

## Abstract

The purpose of this study was to evaluate the predictive features of baseline F-18-fluorodeoxy-D-glucose positron emission tomography (^18^F-FDG PET)/computed tomography (CT) parameters in patients with dedifferentiated liposarcomas (DDLPSs) and well-differentiated liposarcomas (WDLPSs) receiving systemic treatment. A total of 24 patients with liposarcoma who underwent longitudinal ^18^F-FDG PET/CT in systemic therapy were included. All volumetric segmentation of each tumor section and semiquantitative imaging parameters were extracted from the axial field of view from both PET and CT images. Maximum, mean, and minimum standardized uptake values (SUVmax, SUVmean, and SUVmin), Hounsfield units (HUs), and their respective changes from baseline and posttreatment were calculated. The voxel values from unenhanced CT images were correlated with PET-derived parameters. The ^18^F-FDG uptake decreased by more than 56% on average in responders for both SUVmax and SUVmean in DDLPS. There was a decrease in HUmax in DDLPS among responders. Using AUC > 0.8 as a reasonable predictor, we found that the ratios of SUVmaxD/HUmean, SUVmaxD/HUmedian, and SUVmeanD/HUmedian at baseline were significant indicators of the response to treatment in patients with liposarcoma. The changes in SUVmean and not just SUVmax parameters could be considered as accurate tumor response indicators. For the first time, we introduced baseline SUV/HU ratios as a valuable diagnostic tool in predicting liposarcoma treatment outcomes. This ability was not revealed by classic semiquantitative PET or CT parameters at baseline.

## 1. Introduction

Liposarcomas are malignant tumors of adipocytic tissues. As they progress, they become increasingly heterogeneous and exhibit an increase in non-lipomatous components [1]. Tumors can develop in adipose tissues anywhere in the body but are most commonly found in the retroperitoneum and lower extremities, with the majority of cases occurring in adults between the ages of 50 and 65 [2]. The majority of liposarcomas are well-differentiated liposarcomas (WDLPSs) and dedifferentiated liposarcomas (DDLPSs), with WDLPSs representing around 40–50% of liposarcomas and DDLPSs representing 15–20% of cases [3]. Comparing DDLPS to WDLPS, Singer et al. [4] demonstrated that there is a sixfold increase in the risk of death with a dedifferentiated (high-grade) component within the tumor. Therefore, it is extremely important to accurately identify DDLPS prior to initiating treatment owing to the significant differences in prognosis, clinical behavior, and treatment approaches between WDLPS and DDLPS.

There are many challenges associated with treating liposarcoma. Surgical resection remains the primary choice for both DDLPS and WDLPS, and it is associated with a five-year recurrence rate as high as 80% [4]. Our group previously reported that 21% of DDLPS patients have a partial response to chemotherapy and 40% have stable disease following chemotherapy [5]. The heterogeneity of liposarcomas complicates their grading through biopsy. In a study by Ikoma et al. [6], a percutaneous biopsy was found to have a sensitivity of only 36% for DDLPS detection. Hence, novel non-invasive methods for liposarcoma localization, characterization, and treatment evaluation are urgently needed. Positron emission tomography/computed tomography (PET/CT) with [^18^F]-fluorodeoxyglucose (^18^F-FDG) is a unique non-invasive diagnostic tool widely used in the evaluation of numerous malignancies [7,8]. ^18^F-FDG PET/CT is particularly effective in the staging and re-staging of liposarcomas [9,10]. Our group previously concluded in a study of 20 patients that ^18^F-FDG PET/CT is a sensitive and specific diagnostic tool for detecting liposarcomas that have dedifferentiation characteristics [10]. Patients with DDLPS showed significantly higher maximum standardized uptake values (SUVmax) compared to those with WDLPS. Using the SUVmax = 4 cutoff, DDLPSs were identified with a specificity of 85.7% and a sensitivity of 83.3% using ^18^F-FDG PET/CT [10]. Additionally, Li et al. [1] reported an SUVmax of 3.8 as an effective cutoff value for discriminating WDLPS from non-WDLPS, with a median sensitivity of 77% and a specificity of 90%. Wakamatsu et al. [11] further confirmed the poor prognosis associated with high SUVmax values in patients with DDLPS. Patients with DDLPS or grade 2 retroperitoneal sarcomas exhibiting a high SUVmax (>4) had a significantly lower overall survival rate than those with a lower SUVmax (<4) [11]. Baffour et al. [9] demonstrated a correlation between SUVmax and the liposarcoma grade and found overlapping metabolic activity of benign and malignant lipomatous tumors on ^18^F-FDG PET/CT. The morphologic information from CT images also provides significant diagnostic value. For example, lesions with increased metabolic activity of SUVmax > 10 on ^18^F-FDG PET, with an entirely fatty component seen on CT, are pathognomonic of hibernoma. Therefore, considering a combination of PET and CT imaging biomarkers could potentially obviate the need for biopsy.

In this retrospective study, we evaluated the predictive value of quantitative parameters from PET and CT in 24 patients with liposarcoma. The primary aim was to evaluate the changes in ^18^F-FDG uptake in different tumor types at baseline and at the end of treatment. The secondary aim was to assess the predictive value of PET parameters and HU-related parameters from unenhanced CT scans at baseline. Furthermore, we investigated the prognostic values of SUV/HU ratios at baseline and correlated the image texture and its ability to predict the final therapeutic outcome. Regarding SUVmax, which represents uptake in a single voxel, SUVmean and SUVmin parameters reflect the metabolic activity of the entire tumor [12]. Therefore, we also included SUVmean and SUVmin in the analysis.

## 2. Materials and Methods

Through a retrospective review of patients with pathologically confirmed retroperitoneal WDLPS/ DDLPS seen at the University of Texas MD Anderson Cancer Center between June 2012 and August 2022, we identified 24 patients who underwent PET/CT imaging within 3 weeks prior to treatment initiation and underwent available follow-up PET/CT imaging on or after treatment for response evaluation (within 2 months after the last treatment). The presence of a DDLPS component was confirmed pathologically in all cases of DDLPS. We recorded demographic data, including gender, ethnicity, and age, at diagnosis. The response to treatment was recorded per treating physician’s notes, taking into account both the imaging report, SUV avidity, size, and density and the clinical response with improvement in each patient’s symptoms. Currently, there are no valid reproducible response criteria for this disease, and using the treating physician’s notes takes into account both clinical and imaging data. Importantly, all treating physicians in this report were experienced sarcoma medical oncologists.

The study was conducted in accordance with all relevant guidelines and procedures and approved by the University of Texas MD Anderson Cancer Center Institutional Review Board. The informed consent requirement was waived, given the retrospective nature of the study.

PET/CT imaging was performed using the standard institutional protocol. Fasting for at least 6 h was required prior to ^18^F-FDG administration. ^18^F-FDG (approximately a 370 MBq/injection, 10 mCi/injection) was administered intravenously, and patients were asked to rest in a quiet room and instructed to empty their bladder before image acquisition. PET/CT images were acquired at approximately 60 min after ^18^F-FDG administration. Images were acquired on integrated PET/CT scanners (GE Medical System [Milwaukee, WI, USA] or Siemens Medical Systems [Nashville, TN, USA]). Standard vendor-provided reconstruction algorithms were used to reconstruct PET images. CT images were taken from the base of the skull to the proximal thighs at a 3.75 mm slice thickness. Attenuation- and nonattenuation-corrected datasets were reconstructed, and the images were analyzed using MIM version 7.3.3 software (MIM Software Inc., Cleveland, OH, USA).

### 2.1. ^18^F-FDG PET/CT Interpretation and Quantification

Baseline and posttreatment PET/CT images were visually and semiquantitative analyzed by a nuclear medicine physician with more than 20 years of experience. Quantitative interpretation was performed on all tumors, and the volume of interest (VOI) was manually drawn on the whole-body images of the patients, encompassing tumor sites. Analyzing the total tumor volume for DDLPS and WDLPS tumors with ^18^F-FDG PET/CT is challenging due to the size and morphology of these tumors. These tumors were mostly located in the peritoneum, where ^18^F-FDG has significant nonspecific uptake activity in the surrounding tissue, and therefore, any thresholding methods were inadequate in our patient population to achieve a total volume of the tumors, and the corresponding metabolic tumor volume (MTV) and total lesion glycolysis (TLG). Instead, we considered analyzing the VOIs of tumor sections only instead of the full MTVs and manually delineated contours on 3–5 slices in the axial field of view (FOV), where the visually highest uptake was determined at baseline. Representative liver ROIs were recorded. Several semiquantitative features, including SUVmax, SUVmean, and SUVmin, were recorded. As CT images are specified in HUs, the intensity statistics (minimum, maximum, mean, median, and standard deviation values) were also computed in HUs. The histograms from each tumor section of the axial FOV were plotted in HUs, and the mean values were calculated from all textural features.

### 2.2. Statistical Considerations

Statistical analyses were performed using SPSS software (version 24.0; IBM Corp., Armonk, NY, USA). Receiver operating characteristic (ROC) curve analysis was applied to identify and evaluate the diagnostic performance of PET/CT parameters. The area under the curve (AUC) was used to evaluate the accuracy of any prognostic parameters. An AUC higher than 0.8 was arbitrarily considered optimal to predict the final diagnosis. Numeric variables were represented by the mean and the standard deviation (SD). Continuous variables were reported as means ± SDs of SUVmax, SUVmean, SUVmin, and CT HUs. The Kruskal–Wallis, the Mann–Whitney U test, and the paired *t*-test were used to calculate statistical differences between groups, assuming unequal variance, as appropriate. Statistical significance was established for *p*-values of less than 0.05.

## 3. Results

Of the 24 patients included in the study, 12 (50%) were female and 12 (50%) were male, ranging from 41 to 84 years old. All patients were identified as having DDLPS, WDLPS, or a mix of both using ^18^F-FDG PET/CT scans at baseline and underwent follow-up ^18^F-FDG PET/CT within 2 months after the end of treatment. The tumor type was confirmed by histopathology. Patients received a wide variety of systemic treatments, described in detail in Supplementary Table S1. Of those, 8 patients were deemed responders and 16 non-responders based on the treating physician’s notes.

**Figure 1:**
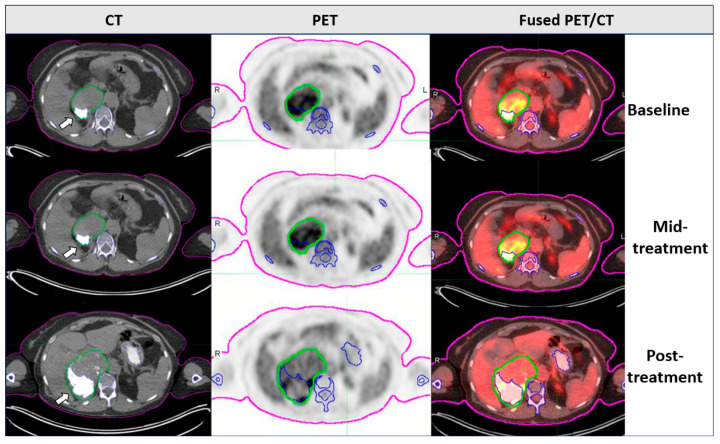
Longitudinal axial CT, PET, and fused PET/CT images demonstrating the presence of ossification (white arrow) in the abdominal DDLPS tumor over the course of treatment. The ossification increased in volume over time; however, the ^18^F-FDG uptake decreased as a treatment response. Since the ossification was invading the tumor volume, this patient was excluded from semiquantitative PET/CT analysis. Delineation color legend: pink = body contour; blue = bone, green = tumor contour.

**Figure 2:**
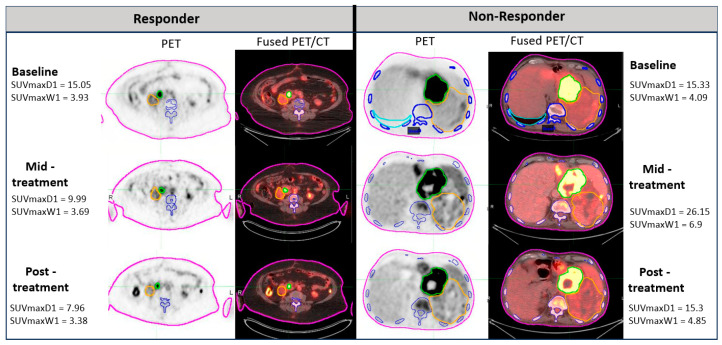
An example of images representing responder and non-responder patients with liposarcoma. Longitudinal axial PET and fused PET/CT images demonstrating the presence of abdominal DDLPS (“D” with green delineation) and WDLPS (“W” with orange delineation) tumors over the course of treatment. The yellow (orange) contours were manually drawn since the threshold segmentation would not pick up the WDLPS due to small differences between the tumor and the background. The DDLPS tumor demonstrated a significant decrease in ^18^F-FDG radiotracer activity (>50% of SUVmax decrease from baseline), as well as in volume in the responder. On the contrary, the DDLPS tumor ^18^F-FDG radiotracer activity increased in the non-responder. In both cases, there was no significant difference in ^18^F-FDG uptake in WDLPS tumors as a response to treatment. Delineation color legend: pink = body contour; blue = bone; sky blue = lung contour.

Of the 24 patients, 2 had a significant ossification component in the DDLPS tumors (1 responder and 1 non-responder). These patients were excluded from quantitative analysis, especially because we focused on identifying PET/CT imaging biomarkers. The ossification in liposarcoma was observed before in our group and has also been described previously by others [13]. Figure 1 describes the baseline, mid-treatment, and posttreatment ^18^F-FDG PET/CT scans of such cases. Ossification of DDLPS leads to a significant increase in density, and while these tumors may respond to treatment, the ossification remains regardless of the treatment response. To obtain a more homogeneous cohort for analysis, we excluded these two cases. Representative images of responder and non-responder patients are depicted in Figure 2. The DDLPS tumor is delineated in green color. The semiautomatic quantification software identified part of the tumor and correlated it to the bone tissue marked in blue color according to the HU values.

### 3.1. ^18^F-FDG PET Metabolic Activity

**Figure 3:**
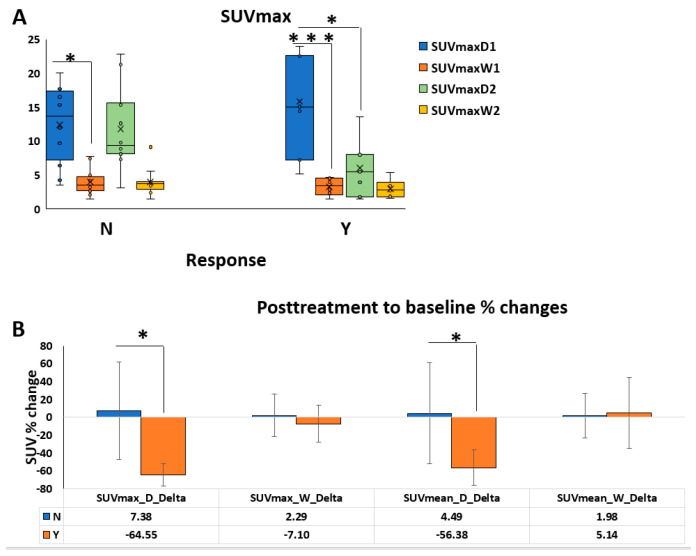
Box-and-whisker plot distribution of semiquantitative PET parameters by histologic subtype. “D” = dedifferentiated liposarcoma (DDLPS); “W” = well-differentiated liposarcoma (WDLPS). The baseline is designated as “1” and the posttreatment value as “2” of the mean value, the standard deviation (SD), the maximum standard uptake value (SUVmax), and the mean standard uptake value (SUVmean). Responders (Y) and non-responders (N). (**A**) SUVmax distribution of DDLPS and WDLPS at baseline (SUVmaxD1/W1) and posttreatment (SUVmaxD2/W2). There was a significant difference in SUVmax between posttreatment and baseline values in responders. The values in the non-responder group did not reach a significant difference. SUVmax values between DDLPS and WDLPS at baseline were significantly different (* *p*-values < 0.05, *** *p*-values < 0.0001). (**B**) SUV percentage change posttreatment compared to baseline values was calculated as “delta” for all relevant parameters. Significant differences were found in SUVmax and SUVmean in DDLPS tumors of responders but not in those of non-responders or in WDLPS tumors in any group.

The mean values of SUVmax, SUVmean, and SUVmin of both tumor types from 22 patients are summarized in Supplementary Table S2. DDLPS tumors had a significantly higher SUVmax at baseline compared to WDLPS tumors, as expected. Conversely, there were no significant SUV differences in both subtypes between responders and non-responders at baseline. The mean SUVmax at baseline for WDLPS (SUVmaxW1) was similar in both groups; (3.27 ± 1.16 for responders vs. 3.77 ± 1.78 for non-responders) and SUVmax at baseline for DDLPS (SUVmaxD1) was not significant either (15.84 ± 7.07 for responders vs. 12.12 ± 5.25 for non-responders) (Figure 3A). As expected, SUVmax significantly decreased by 64.6% in DDLPS responders in posttreatment scans in respect to baseline. However, the SUVmax change in WDLPS was minimal in responders (7% decrease in WDLPS in response to treatment; Figure 3B). SUVmean decreased by 56.4% in DDLPS and increased by 5% in WDLPS in the responder group. Non-responders did not show any significant changes in SUVmax or SUVmean. The SUVmax and SUVmean changes in DDLPS were also found on the ROC curve (AUC = 0.92 and 0.87, respectively) to be effective in detecting posttreatment changes in liposarcoma.

### 3.2. CT Morphologic Features

In our analysis, we compared baseline and posttreatment CT parameters. There were, however, some patients in our cohort who underwent contrast-enhanced CT scans and others who did not. Accordingly, the majority of patients in our non-contrast-enhanced CT cohort underwent the same CT at baseline and posttreatment. Most relevant non-contrast-enhanced CT image parameters from 11 patients are summarized in the Supplementary Figure S1 and Table S2. Liposarcomas have a wide range of CT attenuation (in HUs) within the tumor cross section. Morphologically, WDLPS and DDLPS contain different ratios of tissue composed of fat and a solid mass (identified as areas of dedifferentiation in tumors), where DDLPSs have a higher solid component. Figure S1 in the Supplementary Materials depicts the maximum HU (HUmax) at baseline and after treatment for both subtypes of tumors. No significant differences were seen between responders and non-responders at baseline. The mean HUmax in the DDLPS at baseline in responders was 181.8 ± 153.05, and was 204.4 ± 225.73 in non-responders. Mean HUmax posttreatment values in DDLPS for responders were more uniform (smaller standard deviation of values) compared to non-responders (116.5 ± 25.9 vs. 248.6 ± 256.3, respectively), and HUmax posttreatment values in WDLPS for responders were also more uniform compared to non-responders (107.5 ± 29.48 vs. 322.83 ± 322.4, respectively).

**Figure 4:**
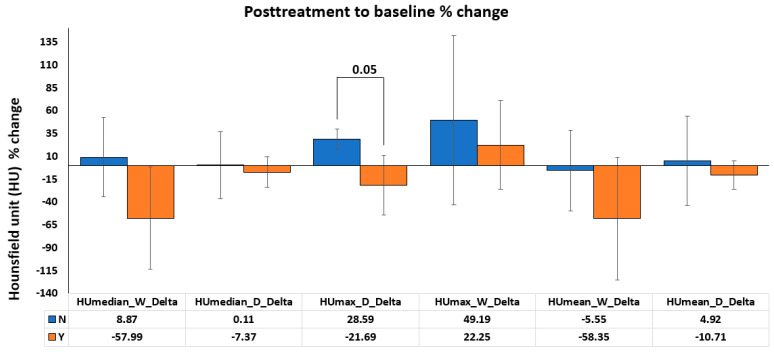
The percentage change between baseline and posttreatment Hounsfield units (HUs). The correlation considered the maximum HU (HUmax) and the median HU (HUmedian) in both subtypes, DDLP and WDLPS.

Our data indicated that there was a decrease in the solid component in both tumor subtypes in the responders’ group on posttreatment CT images, as indicated by decreased HUmax and MaxMeanRatio values (Supplementary Figure S1; Supplementary Table S2). The percentage change between baseline and posttreatment HUmax values in DDLPS responders decreased in comparison to non-responders (*p* = 0.05; Figure 4), suggesting that the decrease in CT attenuation is an important factor in predicting the response to liposarcoma treatments.

**Figure 5:**
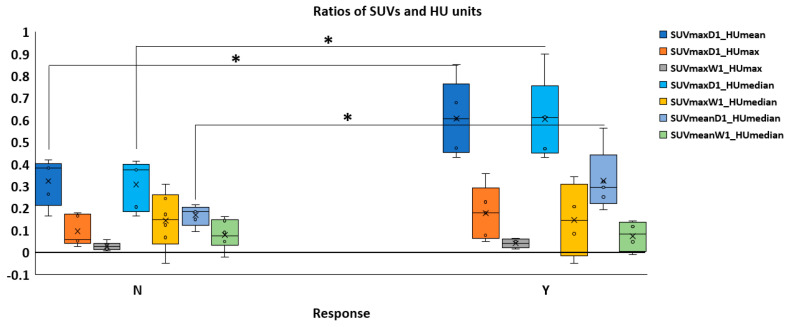
Distribution of the ratios of SUVs and HUs in both subtypes, considering the ratios of SUVmax, SUVmean to HUmax, HUmean, and HUmedian at baseline. Significance (* *p* < 0.05) was achieved in DDLP SUVmaxD1/HUmean, SUVmaxD1/HUmedian, and SUVmeanD1/HUmedian. No significance was found for WDLPS. The baseline is designated as “1” and the posttreatment value as “2.”

**Figure 6:**
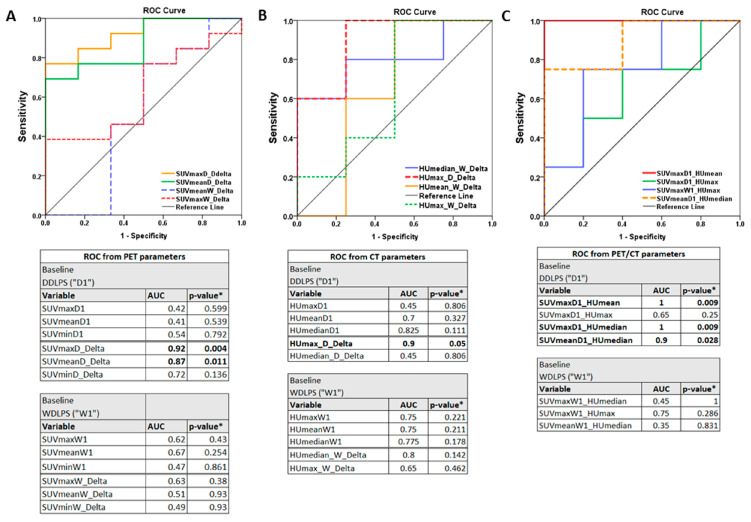
(**A**–**C**) AUCs and *p*-values from ROC curves for semiquantitative PET/CT parameters. An AUC > 0.8 represents the optimal value to predict the outcome of diagnosis. The baseline is designated as “1” and the posttreatment value as “2”. * *p*-values calculated using the AUC under the nonparametric assumption.

Figure 5 demonstrates the SUV/HU ratios in WDLPS and DDLPS. At baseline, SUV/HU ratios were found to be predictive of tumor prognosis for DDLPS according to ROC analysis (Figure 6C). Responders had significantly higher SUV/HU ratios compared to non-responders (*p* < 0.05) for SUVmaxD1 to HUmean (SUVmaxD1/HUmean), SUVmaxD1 to HUmedian (SUVmaxD1/HUmedian), and SUVmeanD1 to HUmedian (SUVmeanD1/HUmedian). The cutoff value for SUVmaxD1/HUmean was >0.52; SUVmaxD1/HUmedian, >0.53; and SUVmeanD1/HUmedian, >0.22. In contrast, the average values for SUVmaxD1/HUmedian for responders compared to non-responders were 0.56 and 0.31, respectively.

## 4. Discussion

This study examined the axial images of all liposarcoma tumors in 24 patients to evaluate the predictive value of semiquantitative ^18^F-FDG PET/CT parameters. We retrospectively analyzed images from different PET/CT scanners; however, the image reconstruction parameters were the same. The results of this study are in accordance with those reported earlier, namely a greater uptake in DDLPS tumors and a significant ^18^F-FDG uptake difference between WDLPS and DDLPS [10,14,15].

In general, a PET scan with ^18^F-FDG is useful to restage disease and assess the response to treatment. It is well recognized that it is difficult to observe a reduction in the uptake and size of tumors following treatment on PET/CT images where the tumor uptake is borderline. Furthermore, tumors with heterogeneous tissue types may exhibit differential sensitivity to chemotherapy and radiation and may not change significantly in size despite effective treatment. In some cases, a tumor may even appear larger in size because of an increase in edema, necrosis, or internal hemorrhage [16]. We examined both baseline and posttreatment image texture changes in conjunction with tumor metabolic activity changes to avoid the aforementioned bias. Ideally, we would want to capture the full metabolic tumor volume (MTV) of all liposarcomas in patients. As ^18^F-FDG has a high non-specific uptake in the peritoneum, any semiautomatic segmentation method would be susceptible to errors and would require manual correction. Therefore, we decided to only analyze a representative section of the tumor on axial PET images, drawing a VOI around the area with the greatest uptake to include the entire lesion on the axial view with several adjacent slices. A representative cross section of the tumor was obtained by this approach, including all cellular components, fat, necrosis, and solid components, and all PET/CT parameters recorded.

Historically, more studies started to include volume-based metabolic parameters, thus obtaining unique metabolic tumor profiles in the total lesion glycolysis (TLG) values of the tumors. TLG is calculated by multiplying the MTV and SUVmean [17]. Choi et al. compared the predictive values of metabolic parameters, including SUVmax, TLG, and the MTV, derived from ^18^F-FDG PET/CT and confirmed that TLG is, indeed, a more accurate predictor of disease progression than SUVmax or MTV CT in soft-tissue sarcomas [18]. Since calculating the true MTV in liposarcoma requires manual correction of segmentation (due to the low difference between tumor and background in some subtypes), it may lead to human error during this process, and such error could propagate into further quantitative analyses. Therefore, we did not include TLG and the MTV in this study.

Wakamatsu et al. [11] investigated how ^18^F-FDG PET can be used as a prognostic tool in the evaluation of prognosis in retroperitoneal sarcoma. The results of this study showed that SUVmax can be used as a prognostic factor in retroperitoneal sarcoma, especially in DDLPS, and SUVmax on an ^18^F-FDG PET scan may be considered for the clinical assessment of a treatment strategy. Here, we were not able to confirm such results from the baseline uptake levels. In our analysis, SUVmax or SUVmean was not significantly different in responders vs. non-responders at baseline for both subtypes. However, as mentioned before, when evaluating PET-based quantification and the subsequent treatment response, it is important to select the appropriate segmentation method. There are many different quantitative thresholding methods used in the literature to classify patients’ responses based on the relative changes in different SUV values [19]. Marles et al. [20] used the semiautomatic segmentation thresholding technique with a threshold equal to 40% of SUVmax. A threshold equal to 40% is usually used in solid tumors [21]; however, this approach was not appropriate for our axial cross-section analysis, especially when the majority of the liposarcomas have fat and potentially necrotic components.

According to a study by Evilevitch et al. [22], reducing metabolic activity as measured by ^18^F-FDG PET/CT is a more accurate indicator of the tumor response than reducing the tumor size as determined by Response Evaluation Criteria In Solid Tumors (RECIST). Schuetze et al. [23] reported about 40% decline in SUVmax in a tumor in which baseline SUVmax was greater than or equal to 6 g/mL, which enabled discriminating responders from non-responders and predicting patients’ outcomes. In our study, SUVmax decreased by 64.6% in DDLPS responders and by 7% in WDLPS responders in response to treatment. SUVmean decreased by 56.4% in DDLPS and by 5% in WDLPS. Non-responders did not display significant changes in SUVmax or SUVmean. We concluded that the SUVmean parameter should be also considered as an accurate indicator of the DDLPS tumor response, as confirmed with ROC curves as well.

We hypothesized that a combination analysis of PET and CT would be useful to determine the efficacy of treatment in patients with WDLPS because ^18^F-FDG uptake in this disease is borderline. First, we investigated whether unenhanced CT attenuations change in response to treatment in liposarcoma. Cihan et al. [24] reported that the higher decrease in CT attenuation is associated with a poor response to chemotherapy in patients with gastric adenocarcinoma. Published data suggest that the mean baseline HU can be used to identify tumor aggressiveness and tumor response in different types of cancer [25,26]. Liposarcomas are fat-containing tumors with a certain degree of solid components (areas of dedifferentiation in tumors); therefore, most of the liposarcomas should have relatively low HU values. Not all patients in our cohort underwent contrast-enhanced CT or were administered contrast consistently at both baseline and on the posttreatment scans. Contrast-enhanced CT images can provide important information about tumor vascularity and perfusion. The use of contrast-enhanced CT imaging may, therefore, become more common. Consequently, further studies should also focus on conducting similar analyses in order to determine whether changes in HU values from contrast-enhanced CT correlate with changes in HU values from non-contrast-enhanced CT. Some reports, however, conclude that radiomic models based on contrast-enhanced CT images do not provide additional value over non-contrast-enhanced CT images [27,28].

As part of our analysis, we compared the most relevant HU-related values obtained from unenhanced CT, and the results resembled those obtained from PET. No significant differences in any HU parameters were found at baseline between WDLPS and DDLPS; however, *p* = 0.05 was achieved when comparing baseline and posttreatment HUmax in DDLPS. HUmax was observed to decrease in WDLPS and DDLPS in responders but not in non-responders. Based on the evidence gathered, we identified some noteworthy findings. It seems that the solid component of the tumor diminishes as a result of the treatment in DDLPS.

Next, we investigated whether the SUV/HU ratio would be a predicting indicator for treatment response as a new diagnostic test for liposarcoma. It is crucial for the evaluation of diagnostic tests’ ability to distinguish the true state of a patient, the determination of the optimal threshold value for each test, and the comparison of two alternative diagnostic tasks when each test is performed on the same individual [29]. ROC curves represent pairs of sensitivity and specificity corresponding to different decision thresholds. The literature suggests that AUC threshold criteria of 0.7 to 0.8 are acceptable, 0.8 to 0.9 are excellent, and more than 0.9 are outstanding [30,31]. In the case of significant findings, we obtained AUCs of 0.9 or greater. The representative cutoffs for the ratios of SUVmaxD1/HUmean (>0.52), SUVmaxD1/HUmedian (>0.53), and SUVmeanD1/HUmedian (>0.22) were significant diagnostic predictors for the response in liposarcoma at baseline (Figure 6). It will be necessary to validate these results with a larger group of patients; however, this is the first report introducing such a ratio as an indicator of the response at baseline in liposarcoma.

There are several limitations of this study. Firstly, this study was performed retrospectively, which may have led to bias. As only unenhanced CT scans were considered in this study, fewer patients were identified for each tumor subtype. Consequently, some groups were relatively small, thereby making it difficult to detect differences between subgroups. Additionally, the group of patients included in this study was heterogeneous, with a wide variety of treatment types. Of note, the relevance of the SUV/HU ratio seems limited to DDLPS only in our study. Finally, since there is a lack of valid reproducible response criteria for DDLPS tumors, the treating physician’s expert opinion at the time of treatment was used to define the response to treatment, which may have introduced some bias.

## 5. Conclusions

Our study suggests that SUV/HU ratios may be useful in predicting the outcome of patients receiving treatment for DDLPS. This ability was not revealed at baseline by the traditional semiquantitative PET/CT parameters. Larger cohorts are required to confirm our findings.

## Data Availability

The data presented in this study are available upon request from the corresponding author.

## Supplementary Materials

The following supporting information can be downloaded at https://www.mdpi.com/article/10.3390/diagnostics14182021/s1, Table S1: The median treatment duration monitored by PET/CT imaging was 15.5 weeks (up to 46 weeks); Table S2: Summary of the metabolic activity of the liposarcomas at baseline and the last post-treatment; Figure S1: Representative HU values in responders (Y) vs non responders (N).

## Abbreviations

DDLPS	dedifferentiated liposarcoma
WDLPS	well-differentiated liposarcoma
SD	standard deviation
SUVmax	maximum standard uptake value
SUVmean	mean standard uptake value
SUVmin	minimum standard uptake value
Y	responders
N	non-responders
Delta	percentage change posttreatment compared to baseline
HUmax	maximum Hounsfield units
HUmean	mean Hounsfield units
HUmin	minimum Hounsfield units
HUmedian	median Hounsfield units
ROC	receiver operating characteristic
AUC	area under the curve
