# Supplementary material for: Predictive Value of Quantitative Parameters of 18F-FDG PET/CT in Patients with Liposarcoma
Source: Diagnostics (Basel). 2024 Sep 12;14(18):2021. doi: 10.3390/diagnostics14182021 (PMC11431839; doi:10.3390/diagnostics14182021)
Supplement: Supplementary file 1 [file diagnostics-14-02021-s001.zip › diagnostics-3141778-supplementary.pdf]

**Supplementary Table S1.** The median treatment duration monitored by PET/CT imaging was 15.5 weeks (up to 46 weeks). We observed 71% of responders exhibited significant SUVmax modification on the first available post-treatment PET/CT (a median of 7 weeks, range 5-19 weeks).

| | Histology | Treatment | Duration (weeks) | Response (YES/NO) | Notes per treating physicians |
|---|---|---|---|---|---|
| 1 | DDLPS/WDLPS | Gem/Tax | 12 | NO | Mixed response after 2 cycles, PD after 4 cycles |
| 2 | DDLPS/WDLPS | Doxil | 9 | NO | Slow progression |
| 3 | DDLPS/WDLPS | Eribulin | 6 | NO | Progression |
| 4 | DDLPS/WDLPS | Gem/Tax | 14 | YES | Response, ossification component |
| 5 | DDLPS/WDLPS | Palbo | 3 | NO | Progression |
| 6 | DDLPS/WDLPS | ADTIC | 20 | YES | Response |
| 7 | DDLPS/WDLPS | Palbo | 43 | YES | Response |
| 8 | DDLPS/WDLPS | Immunotherapy | 8 | NO | Slight progression |
| 9 | DDLPS/WDLPS | Palbo | 15 | NO | Stable |
| 10 | WDLPS | Gem/Tax | 20 | NO | Stable |
| 11 | DDLPS/WDLPS | Gem/Tax | 8 | NO | Progression |
| 12 | DDLPS/WDLPS | Adriamycin | 21 | YES | Response |
| 13 | DDLPS/WDLPS | Selinexor | 17 | NO | Progression |
| 14 | DDLPS/WDLPS | Palbo | 9 | NO | Progression |
| 15 | DDLPS/WDLPS | Palbo | 39 | NO | Progression, ossification component |
| 16 | DDLPS/WDLPS | AI | 16 | NO | Progression |
| 17 | WDLPS | Palbo | 28 | NO | Stable and then progression |
| 18 | DDLPS/WDLPS | Adria-Olara | 14 | YES | Response |
| 19 | DDLPS | ADTIC | 7 | NO | Progression |
| 20 | DDLPS/WDLPS | AI | 36 | YES | Response |
| 21 | DDLPS/WDLPS | Gem/Tax | 30 | NO | Stable |
| 22 | DDLPS/WDLPS | Gem/Tax | 39 | YES | Response |
| 23 | DDLPS/WDLPS | Gemcitabine | 46 | NO | Low progression |
| 24 | DDLPS/WDLPS | Gem/docetaxe | 9 | YES | Response |

**Abreviation**: Gem/Tax = gemcitabine (Gemzar) and paclitaxel (Taxol); Doxil = Liposomal Doxorubicin; Palbo = Palbociclib; Adriamycin = Doxorubicin; ADTIC = adriamycin + dacarbazine + tremelimumab + ifosfamide + carboplatin; AI = adriamycin + ifosfamide; Adria-Olara = adriamycin + olaratumab; Gemcitabine and docetaxel
PD = Progressive Disease.

**Supplementary Table S2.** Summary of the metabolic activity of the liposarcomas at baseline and the last post-treatment. $^{18}$F-FDG PET/CT scans in well-differentiated liposarcoma (WDLPS) and dedifferentiated liposarcoma (DDLPS) tumors; Semiquantitative PET parameters derived from tumors are designated as "D" for DDLP, and "W" for WDLPS. Baseline is designating with '1" and post treatment values as "2" of Mean value, Standard deviation (SD), maximum Standard Uptake Value (SUVmax), mean Standard Uptake Value (SUVmean), minimum Standard Uptake Value (SUVmin), and representative p-values. Percent change of posttreatment to baseline values were calculated as "Delta" for all relevant parameters. All the other abbreviations can be found in the Abbreviations.

**Baseline DDLPS ("D1")**

| Variable | Responders | | Non responders | | |
|---|---|---|---|---|---|
| **PET** | **Mean** | **Standard deviation** | **Mean** | **Standard deviation** | **p-values*** |
| SUVmaxD1 | 15.84 | 7.07 | 12.12 | 5.25 | 0.322 |
| SUVmeanD1 | 8.60 | 3.70 | 7.05 | 2.55 | 0.285 |
| SUVminD1 | 2.95 | 1.72 | 2.79 | 1.41 | 0.78 |
| **SUVmaxD_Delta** | **-64.55** | **12.70** | **7.38** | **54.37** | **0.002** |
| **SUVmeanD_Delta** | **-56.38** | **20.16** | **4.49** | **56.77** | **0.005** |
| SUVminD_Delta | -24.46 | 64.79 | 4.58 | 61.42 | 0.075 |
| **CT (in HU units)** | **Mean** | **Standard deviation** | **Mean** | **Standard deviation** | **p-values*** |
| HUmaxD1 | 181.80 | 153.05 | 204.40 | 225.73 | 0.92 |
| HUmedianD1 | 35.00 | 10.49 | 36.60 | 10.09 | 0.4 |
| MaxMeanRatioD1 | 0.98 | 1.06 | 0.53 | 0.62 | 0.68 |
| **HUmax_D_Delta** | **-21.69** | **32.48** | **28.59** | **10.77** | **0.05** |
| HUmedian_D_Delta | -7.37 | 16.70 | 0.11 | 37.00 | 0.806 |
| HUmean_D_Delta | -10.71 | 15.40 | 4.92 | 48.74 | 1 |

**Posttreatment DDLPS ("D2")**

| Variable | Responders | | Non responders | | |
|---|---|---|---|---|---|
| **PET** | **Mean** | **Standard deviation** | **Mean** | **Standard deviation** | **p-values*** |
| **SUVmaxD2** | **6.00** | **3.93** | **11.49** | **5.55** | **0.016** |
| **SUVmeanD2** | **3.71** | **1.96** | **6.43** | **2.50** | **0.029** |
| SUVminD2 | 1.95 | 1.38 | 2.46 | 1.28 | 0.251 |
| **CT (in HU units)** | **Mean** | **Standard deviation** | **Mean** | **Standard deviation** | **p-values*** |
| HUmaxD2 | 116.50 | 25.87 | 248.60 | 256.34 | 0.624 |
| HUmedianD2 | 28.75 | 7.00 | 33.40 | 9.07 | 0.539 |
| MaxMeanRatioD2 | 0.02 | 0.40 | 0.08 | 3.00 | 0.806 |

**Baseline WDLPS ("W1")**

| Variable | Responders | | Non responders | | |
|---|---|---|---|---|---|
| **PET** | **Mean** | **Standard deviation** | **Mean** | **Standard deviation** | **p-values*** |
| SUVmaxW1 | 3.27 | 1.16 | 3.77 | 1.78 | 0.64 |
| SUVmeanW1 | 1.62 | 0.64 | 1.97 | 0.80 | 0.312 |
| SUVminW1 | 0.73 | 0.38 | 0.67 | 0.40 | 0.755 |
| SUVmaxW_Delta | -7.10 | 20.43 | 2.29 | 23.80 | 0.312 |
| SUVmeanW_Delta | 5.14 | 39.91 | 1.98 | 25.00 | 0.876 |

| | | | | | |
|---|---|---|---|---|---|
| SUVminW_Delta | -8.01 | 21.75 | -9.91 | 28.33 | 0.876 |
| **CT (in HU units)** | **Mean** | **Standard deviation** | **Mean** | **Standard deviation** | **p-values*** |
| HUmaxW1 | 93.25 | 22.99 | 195.67 | 178.47 | 0.52 |
| HUmedianW1 | -10.25 | 46.09 | 14.67 | 24.86 | 0.34 |
| MaxMeanRatioW1 | 0.82 | 1.05 | 0.65 | 0.49 | 0.08 |
| HUmax_W_Delta | 22.25 | 48.54 | 49.19 | 92.70 | 0.52 |
| HUmedian_W_Delta | -57.99 | 56.02 | 8.87 | 43.35 | 0.088 |
| HUmean_W_Delta | -58.35 | 67.29 | -5.55 | 44.06 | 0.286 |

| **Posttreatment WDLPS ("W2")** | | | | | |
|---|---|---|---|---|---|
| **Variable** | **Responders** | | **Non responders** | | |
| **PET** | **Mean** | **Standard deviation** | **Mean** | **Standard deviation** | **p-values*** |
| SUVmaxW2 | 2.96 | 1.23 | 3.69 | 1.73 | 0.243 |
| SUVmeanW2 | 1.60 | 0.51 | 1.90 | 0.65 | 0.392 |
| SUVminW2 | 0.63 | 0.24 | 0.60 | 0.38 | 0.392 |
| **CT (in HU units)** | **Mean** | **Standard deviation** | **Mean** | **Standard deviation** | **p-values*** |
| HUmaxW2 | 107.50 | 29.48 | 322.83 | 322.40 | 0.67 |
| HUmedianW2 | -9.50 | 26.13 | 7.83 | 37.69 | 0.28 |
| MaxMeanRatioW2 | 0.25 | 0.06 | 0.96 | 0.73 | 0.14 |

* p-values between-group comparisons of continuous data categorical variables were calculated from using the Kruskal-Wallis test in respect to a treatment response. Statistical significance was established for p-values of less than 0.05.

## Supplementary Figure S1

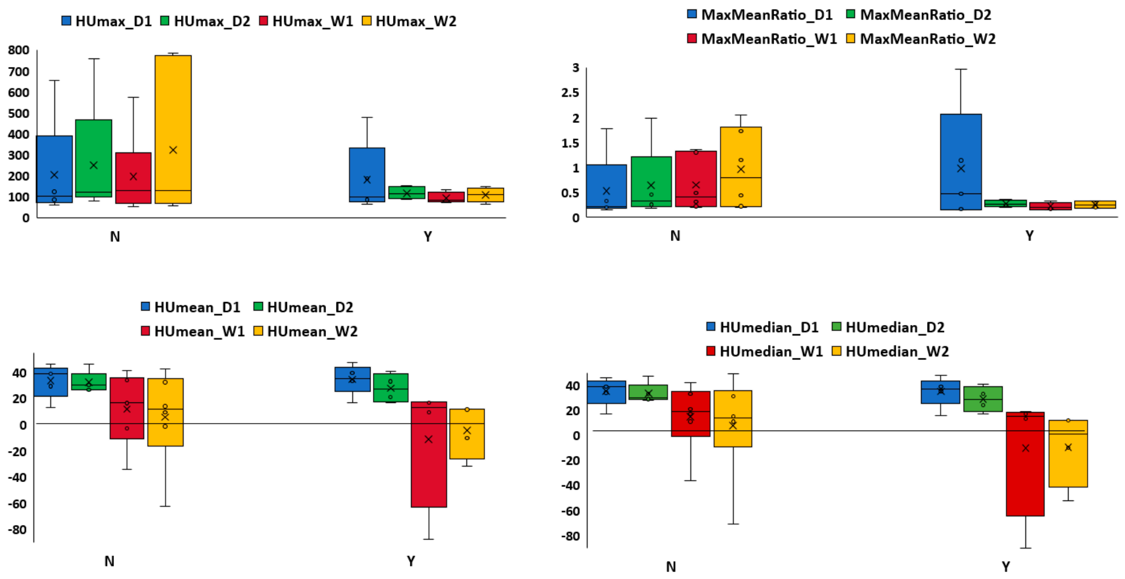


**Figure S1**: Representative HU values in responders (Y) vs non responders (N). HUmax and MaxMeanRatios follow the same patterns, where HU values in responders are lower compared to the non-responder counterparts. HUmean and HUmedian did not reveal any differences and baseline or posttreatment. Baseline is designating with ‘1” and post treatment values as “2”. All the other abbreviations can be found in Abbreviations.
